# Characterization of protein adsorption onto FePt nanoparticles using dual-focus fluorescence correlation spectroscopy

**DOI:** 10.3762/bjnano.2.43

**Published:** 2011-07-12

**Authors:** Pauline Maffre, Karin Nienhaus, Faheem Amin, Wolfgang J Parak, G Ulrich Nienhaus

**Affiliations:** 1 Institute of Applied Physics and Center for Functional Nanostructures, Karlsruhe Institute of Technology (KIT), Wolfgang-Gaede-Straße 1, 76131 Karlsruhe, Germany; 2 Department of Physics, Philipps University Marburg, Renthof 7, 35037 Marburg, Germany,; 3 Department of Physics, University of Illinois at Urbana-Champaign, 1110 West Green Street, Urbana, IL 61801, USA

**Keywords:** apolipoprotein, dual-focus fluorescence correlation spectroscopy, human serum albumin, nanoparticle, protein adsorption

## Abstract

Using dual-focus fluorescence correlation spectroscopy, we have analyzed the adsorption of three human blood serum proteins, namely serum albumin, apolipoprotein A-I and apolipoprotein E4, onto polymer-coated, fluorescently labeled FePt nanoparticles (~12 nm diameter) carrying negatively charged carboxyl groups on their surface. For all three proteins, a step-wise increase in hydrodynamic radius with protein concentration was observed, strongly suggesting the formation of protein monolayers that enclose the nanoparticles. Consistent with this interpretation, the absolute increase in hydrodynamic radius can be correlated with the molecular shapes of the proteins known from X-ray crystallography and solution experiments, indicating that the proteins bind on the nanoparticles in specific orientations. The equilibrium dissociation coefficients, measuring the affinity of the proteins to the nanoparticles, were observed to differ by almost four orders of magnitude. These variations can be understood in terms of the electrostatic properties of the proteins. From structure-based calculations of the surface potentials, positively charged patches of different extents can be revealed, through which the proteins interact electrostatically with the negatively charged nanoparticle surfaces.

## Introduction

Recent years have seen enormous advances in the field of nanotechnology. A huge variety of nanoparticles (NPs), defined as objects with all three spatial dimensions in the range of 1–100 nm, has been developed, with well-controlled physicochemical properties including size, shape, charge, chemical composition and solubility. Many of these NPs have already found their way into consumer products.

Owing to their small size, NPs may potentially invade all parts of the human body including tissues, cells and even subcellular compartments. Consequently, they hold great promise as tools for biomedical applications such as targeted drug delivery [1] or gene therapy [2]. However, NPs often exhibit properties distinctly different from those of the bulk material. For example, an enhanced surface reactivity may be observed due to their large surface-to-volume ratio [3] and, therefore, NPs may also pose a biological hazard [4–5].

Upon incorporation into the body, NPs become exposed to biological fluids such as lung epithelial lining fluid or blood plasma, which contain a variety of dissolved molecules, especially proteins. Depending on the properties of its surface, a NP may adsorb proteins and other biomolecules from the fluid to a lesser or greater extent. A protein coating layer, the so-called ‘protein corona’, forms and can completely enshroud the NP [6–11]. Consequently, at least the initial encounter of a NP with a cell is governed by the properties of the protein corona rather than those of the NP surface [12]. NP–protein interactions are typically weaker than chemical bonds and still comparable to the thermal energy at physiological temperatures. Therefore, the protein corona is not static but fluctuates in time due to incessant protein association and dissociation events. Upon biofluid exposure, the NP surface will quickly become coated with those proteins that are prevalent in the fluid and that have high binding rate coefficients. However, these proteins may subsequently be replaced by less prevalent proteins with higher binding affinity. Eventually, equilibrium will be established, so that the relative abundance of proteins in the corona is determined by their binding strength to the NP and their concentrations in the biofluid. We note that this simple equilibrium binding model is likely an oversimplification that needs further elaboration because proteins are complex physical systems that can assume a large number of different conformations [13–14]. The net free energies involved in NP–protein interactions can match or even exceed the entire internal stabilization energy of proteins. Their structures may change upon contact with a NP surface, up to the point that they entirely unfold. Such effects are known from the development of nanostructured surface coatings designed to prevent unspecific biomolecular adsorption (‘biofilms’) [15–17], which is an important issue for various fields including biotechnology (e.g., biosensors, bioanalytics) and biomedical devices (e.g., implants and catheters).

To be able to control the biological effects of NPs, such as prevention of uptake or targeted delivery to specific cells or tissues, it is of utmost importance to understand the structural and dynamic properties of the protein corona at the molecular level. Recently, we have used quantitative fluorescence microscopy, especially fluorescence correlation spectroscopy (FCS), to study protein adsorption of human serum albumin (HSA) on polymer-coated FePt NPs with an overall diameter of 11 nm [11]. HSA is the major soluble constituent of human blood plasma. It serves primarily as a carrier protein for steroids, fatty acids, and thyroid hormones [18]. We found that, at concentrations typically found in blood serum, ~20 HSA molecules adsorb as a monolayer of ~3.3 nm thickness on these NPs, and time-resolved fluorescence quenching experiments revealed a typical protein residence time of ~100 s [11]. For transferrin [8], an important blood plasma protein involved in iron transport and delivery, we observed formation of a 7 nm thick protein corona.

The FCS method is based on the analysis of the duration of brief bursts of photons from individual fluorescence emitters, diffusing through an observation volume of about 1 fL in a confocal microscope [19–23]. Autocorrelation analysis of the fluorescence intensity time traces yields the characteristic time scale of diffusion, *τ*_D_. Based on the well-known spatial extension of the observation volume, the diffusion coefficient, *D*, and, by using the Stokes–Einstein equation (see Experimental), the hydrodynamic radius of the fluorescent particle, *R*_H_, can be calculated. Consequently, a NP size increase due to protein adsorption onto the NP surfaces can be measured via an increase of *τ*_D_. Knowledge of the molecule detection function (MDF), i.e., the probability to detect a fluorescence photon from a molecule at a given position in the sample volume, is key to the precise quantitative analysis of an FCS experiment [24]. The MDF depends on the intensity distribution of the focused laser beam used for excitation, the distribution of detection efficiencies of photons emanating from the observation volume and the photophysical properties of the fluorophores. It is sensitive to various parameters of the optical setup, including the refractive index mismatch between the sample solution and the immersion medium, variations in cover-slide thickness and astigmatism of the laser beam. Only by extremely careful calibration procedures and measurements can the subnanometer precision required for studying protein adsorption on NPs be achieved.

Dual-focus FCS (2fFCS) is a variant of the FCS method that includes an absolute calibration standard and promises to make high-precision particle size measurements much easier [25]. In 2fFCS, two laterally shifted, partially overlapping laser foci are positioned in the sample at a known, fixed separation. (Further details are given in Experimental.) Accurate diffusion coefficients can be obtained by a combined (‘global’) analysis, for each of the two detection volumes, of the autocorrelation function of the photon arrival times, i.e., the probability to detect a photon at time, *t* + *τ*, given that a photon was detected at time *t*, and the cross-correlation between the two volumes, i.e., the probability to detect a photon from one volume at *t* + *τ*, given that a photon was detected in the other volume at time *t*.

Here we have employed the 2fFCS method to quantify the equilibrium binding of three abundant blood plasma proteins to FePt NPs, HSA (which was included to ensure that our previously reported data [11] can be reproduced with our new technique) and the apolipoproteins apoA-I and apoE4. These two proteins function as transporters for lipid molecules in the blood by binding a large number of lipid and cholesterol molecules to form water-soluble lipoproteins, and they direct the lipids to their correct destinations in the body [26–28].

## Results and Discussion

### Protein equilibrium binding to FePt NPs

For studying the interaction of serum proteins with NPs by 2fFCS, we employed the same type of NP as in our previous work [11], namely, FePt cores that were rendered fluorescent by incorporating a small number of red fluorescent dye molecules (DY-636) in the polymer-coating surrounding the core [29]. The polymer shell contained a large number of carboxyl groups endowing the NPs with an overall negative charge and excellent colloidal stability [30].

**Figure 1:**
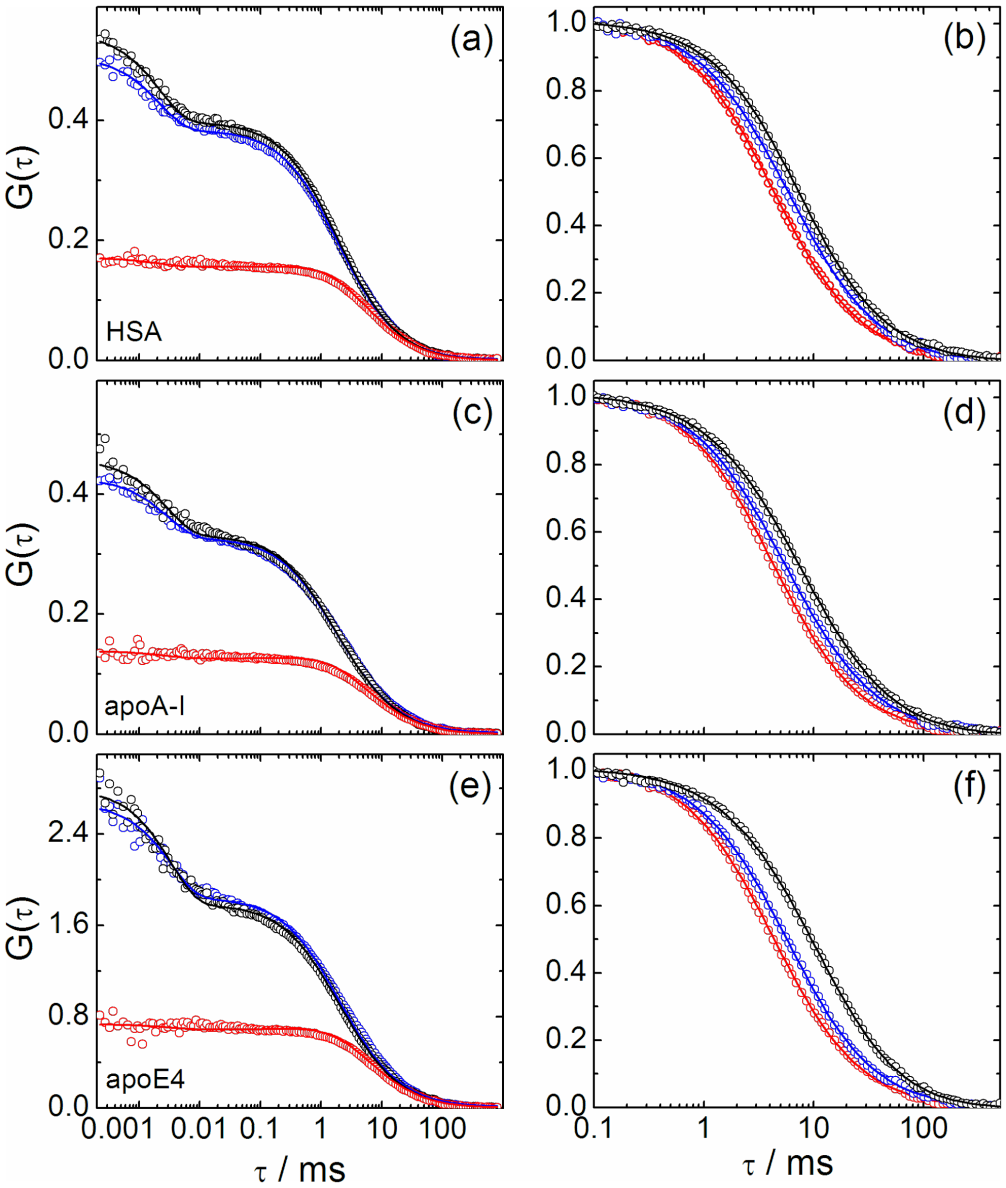
Fluorescence intensity correlation curves of NPs dissolved in buffer solutions of (a, b) HSA, (c, d) apoA-I, and (e, f) apoE4. (a, c, e) Measured (symbols) and fitted (lines) 2fFCS autocorrelation (black and blue) and cross-correlation (red) functions of polymer-coated FePt NPs in the presence of (a) 400 µM HSA, (c) 285 µM apoA-I and (e) 7.2 µM apoE4. (b, d, f) Measured (symbols) and fitted (lines) cross-correlation curves of NPs in buffer solution (red) and in the presence of serum proteins at two concentrations, normalized to 1 at *τ* = 0.1 ms. (b) 6.3 and 400 µM HSA (blue, black); (d) 36 and 285 µM apoA-I (blue, black); (f) 14 nM and 7.2 µM apoE4 (blue, black).

To determine the affinity of the proteins to the NPs as well as the increase in *R*_H_, we took 2fFCS data on NPs freely diffusing in solutions, which contained the proteins at concentrations varying over several orders of magnitude. NP concentrations in the nanomolar range ensured that roughly only one NP resided in the detection volume on average, so that the intensity fluctuations, on which the FCS method is based, were large. The protein concentration was varied on a logarithmic scale in a selected range appropriate for observing the transition from uncoated to coated NPs. Examples of measured correlation curves are depicted in Figure 1 for HSA, apoA-I and apoE4 (top to bottom). In the left column, representative correlation curves are shown at one selected protein concentration, i.e., autocorrelation curves for the two foci and the cross-correlation curve. Note that FCS data and, therefore, also the derived *R*_H_ values, are averages determined from a few thousand single-particle bursts. The autocorrelation curves in Figure 1 display two decay processes. The step on the millisecond time scale is due to NP diffusion and, therefore, reveals the particle size, whereas the step on the microsecond time scale arises from dye photophysics and is not of interest here. It originates from interconversion to the triplet state; fluorophores cease to emit fluorescence until they return to the ground state and can be excited again. Note that this process is strongly suppressed in the cross-correlation function because of its short time scale and the small overlap of the two detection volumes.

In the right column of Figure 1, cross-correlation curves are plotted for different protein concentrations in the solution, normalized to 1 at τ = 0.1 ms (for ease of comparison). Evidently, the curves shift toward longer times with increasing protein concentration, indicating that the effective size of the NPs grows due to protein adsorption. The effect is small, however, so precise data are needed for a quantitative analysis of protein binding.

**Figure 2:**
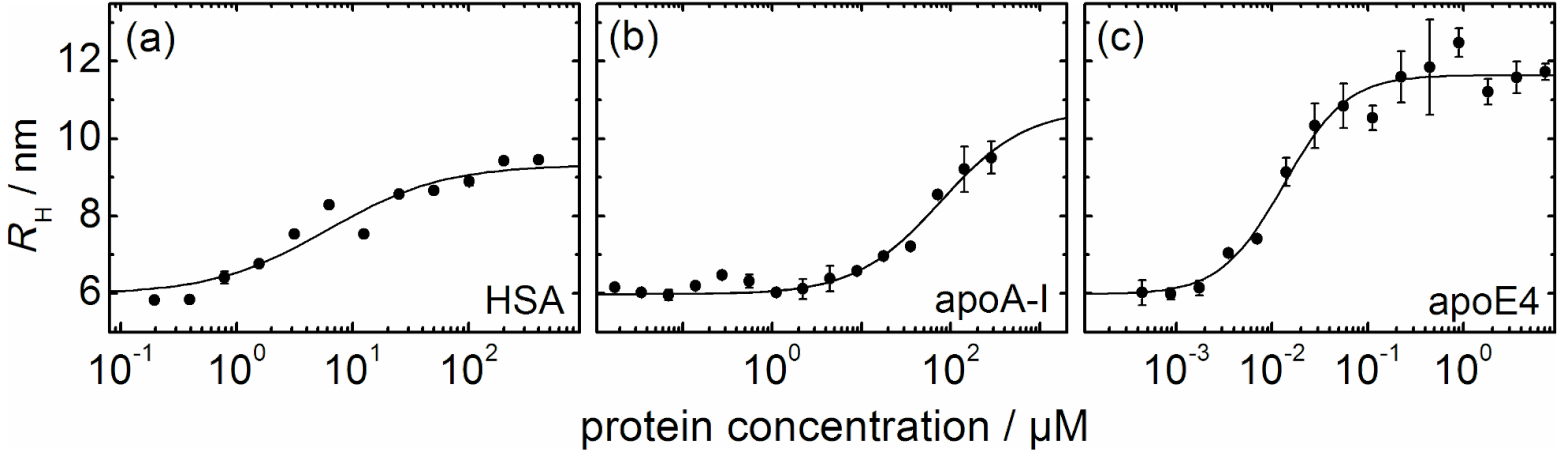
Hydrodynamic radius *R*_H_, of the FePt NPs, plotted as a function of the concentration of (a) HSA, (b) apoA-I and (c) apoE4. The curves (solid lines) were fitted according to Equation 1 and Equation 2; best-fit parameters are compiled in Table 1.

Figure 2 shows the dependence of *R*_H_ on the logarithm of the protein concentration, as obtained from the 2fFCS correlation data. For all three proteins, *R*_H_ increases in a stepwise fashion with protein concentration, as we previously reported for HSA and transferrin [8,11], which indicates a limited loading capacity of the NPs. This behavior can be understood if we assume that the protein molecules form a monolayer around the NPs, with a well-defined thickness, Δ*R*_H_, and binding affinity, *K’*_D_, as quantified by the protein concentration at the midpoint of the binding transition (vide infra). Once the monolayer is formed, the NP size remains constant, and the tendency to further accrete protein is essentially zero.

The data in Figure 2 can be analyzed quantitatively by using the following model. The hydrodynamic radius, *R*_H_, of a spherical object is given by

[3]
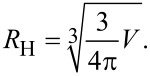


Consequently, we can express the dependence of *R*_H_ on the number of bound proteins by

[4]
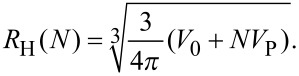


Note that we make the assumption that the protein-coated NP can still be well approximated by a sphere. In Equation 4, *V*_0_ is the volume of the NP, and *N* is the number and *V*_P_ the molecular volume of the adsorbed proteins. (Proteins have a typical density of 1.35 g/mL, so their volume is, to a good approximation, proportional to their mass). By introducing the radius of the bare NP, *R*_H_(0), and the coefficient *c* = *V*_P_/*V*_0_,

[1]
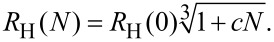


Consequently, upon complete formation of the protein corona,

[5]
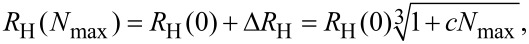


where the maximum number of proteins binding to the NP is denoted by *N*_max_. We model the dependence of *N* on the concentration of free protein, [*P*], by the Hill equation [11],

[2]
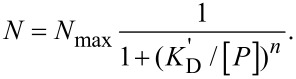


Here, the equilibrium dissociation coefficient, *K’*_D_, denotes the midpoint of the transition, i.e., the concentration of protein molecules free in the solution at half coverage. It quantifies the strength of the NP–protein interaction. The Hill coefficient, *n*, controls the steepness of the curve; it contains information about the cooperativity of binding. The lines in Figure 2 represent fits of Equations 3 and 5 to the data. Because all the FePt NPs were from the same batch, their hydrodynamic radius, *R*_H_(0) = (6.0 ± 0.1) nm, was taken as a global parameter in the fit for all three proteins. The best-fit parameters in Table 1 will be discussed in relation to the molecular structures of the proteins in the following subsections.

**Table 1:** Parameters of protein adsorption onto FePt NPs.

Protein	*R*_H_(*N*_max_) (nm)	Δ*R*_H_ (nm)	*K’*_D_ (µM)	*n*	*N*_max_

HSA	9.3 ± 0.3	3.3 ± 0.3	9.9 ± 4.7	0.9 ± 0.2	27 ± 3
ApoA-I	10.8 ± 1.5	4.8 ± 1.4	140 ± 60	1.0 ± 0.3	52 ± 10
ApoE4	11.7 ± 0.3	5.7 ± 0.2	0.021 ± 0.003	1.4 ± 0.2	65 ± 3

### Structure of the protein corona

Comparison of the data in Figure 2 shows that the thickness of the protein corona, Δ*R*_H_, is a characteristic of the particular protein species adsorbed. In our previous studies with HSA [11] and transferrin [8], we noticed that the thickness of the protein corona was correlated with the molecular dimensions of the proteins as obtained from the X-ray structures. These observations gave additional support to our claim that the corona consists of a monolayer of proteins adsorbed in specific orientations. Considering the strengths of Coulombic interactions, the molecular orientations are likely to be governed by patches of positive surface charge on the protein that preferentially interact with the negatively charged NP surface. In this subsection, we discuss the thickness of the corona in relation to the molecular shapes and electrostatic properties of the adsorbed proteins.

**Figure 3:**
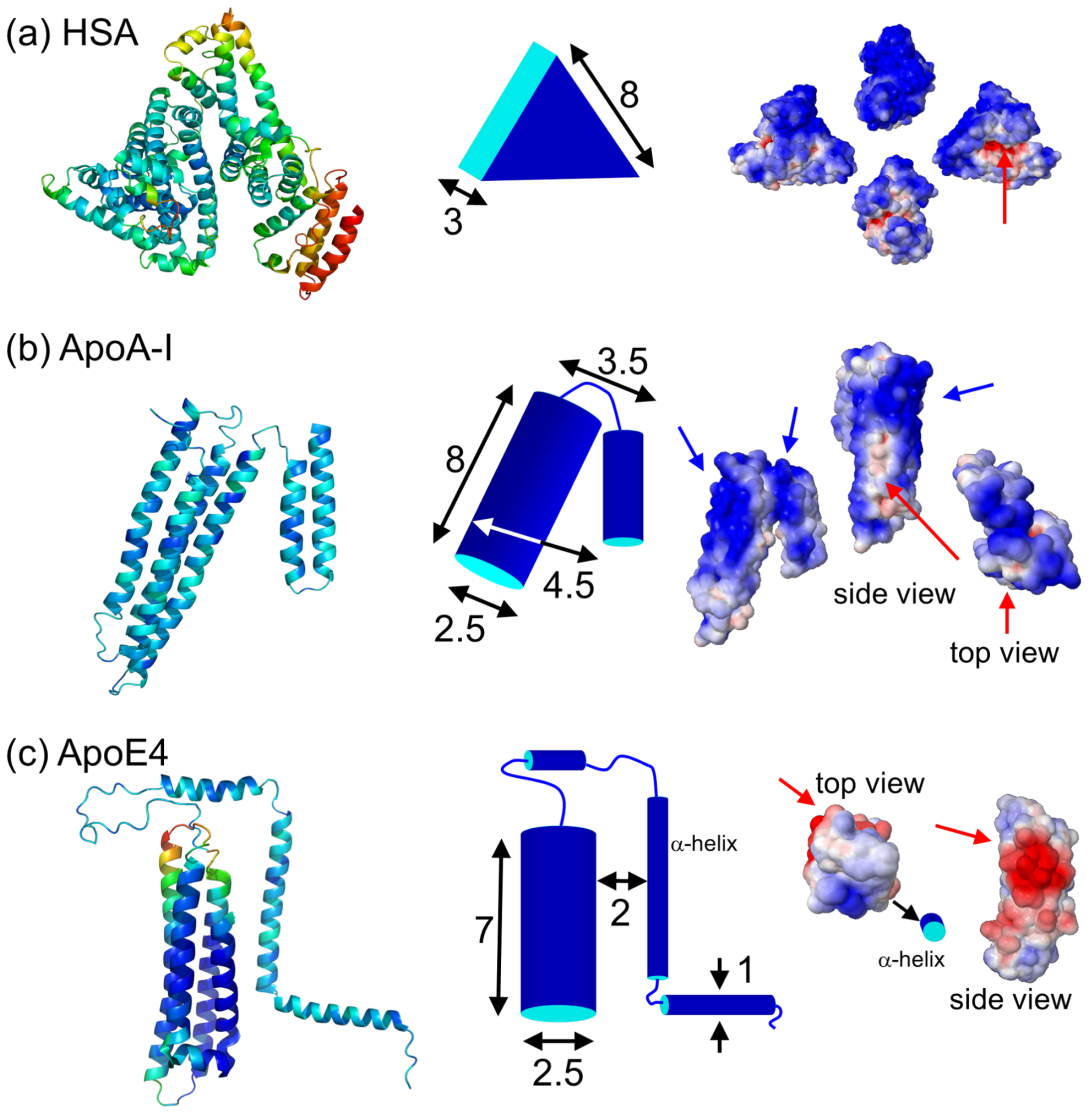
Structural depictions of (a) HSA, (b) apoA-I and (c) apoE4. Left column: Cartoon representations of HSA (protein data bank accession (pdb) code 1AO6), apoE4 (pdb code 1GS9) and apoA-I (pdb code 2A01). For apoE4, only the structure of the 22-K domain (4-helix bundle) has been solved. Center column: Simplified representations of the proteins including approximate dimensions (in nm). Right column: Space-filling models colored to indicate their surface electrostatics at pH 7.4 (blue: negative potential, red: positive potential; range from −5 *k*_B_T/*e* to +5 *k*_B_T/*e*; calculated online at http://kryptonite.nbcr.net/pdb2pqr/ [31]).

Figure 3a (left) shows a cartoon representation of the molecular structure of HSA, a protein with a molecular mass of 67 kDa [18]. It can be approximated by an equilateral triangular prism, with sides of ~8 nm and a height of ~3 nm (Figure 3a, middle). The ~3 nm radius increase upon adsorption of HSA, observed with 2fFCS (Figure 2a), completely agrees with our previous data [11], which led us to the suggestion that HSA molecules adsorb via their triangular surfaces onto the NPs. Also shown in Figure 3a (right) are space-filling models colored so as to visualize the electrostatic surface potentials. One of the triangular protein surfaces shows a pronounced positive patch, which is likely to promote the interaction with the negatively charged carboxyl groups on the NP surfaces (red arrow, Figure 3a). Overall, about 27 HSA molecules fit into the volume generated by the size increase of the NP (Table 1).

Lipid-free human apoA-I is the principal component of high-density lipoprotein (HDL) and plays an essential role in lipid transport and metabolism. This protein has a molecular mass of 28 kDa. X-ray crystallography revealed a two-domain structure, with a N-terminal domain forming a four-helix bundle and a structurally less well organized C-terminal domain (Figure 3b, left) [32–34]. In solution, apoA-I appears to be more flexible than in the crystalline state [35–36]. Based on analytical ultracentrifugation, viscometric, and fluorescence studies, its overall shape has been described by a prolate ellipsoid with an axial ratio of 5.5:1 [37–38]. Due to mutual interactions, the C-terminal domain is kept in close proximity to the N-terminal helix bundle, contributing significantly to the stability of the lipid-free conformation [39]. Förster resonance energy transfer (FRET) studies have indicated that the inter-domain distance in solution is even smaller than in the crystal structure [39]. Figure 3b (middle) gives a crude depiction of the structure of apoA-I in solution. On its surface, there are two rather extended negatively charged patches (marked by blue arrows in Figure 3b, right) that have been associated with the recognition of the ATP-binding cassette transporter A1 (ABCA1) [34]. In close vicinity to the larger patch, a small area of positive electrostatic potential is visible, which would be favorable for the interaction with our negatively charged NPs (red arrows in Figure 3b). By attaching with this patch to the NP surface, the apoA-I molecules are expected to form a layer of ~4–5 nm thickness, which is in good agreement with our experimental findings (Figure 2b). The protein corona consists of on the order of 50 apoA-I molecules (Table 1).

Human apoE4 is another member of the family of soluble apolipoproteins [26]. The 34 kDa protein preferentially binds to very low-density lipoprotein (VLDL) and intermediate-density lipoprotein and has a high affinity for the low-density lipoprotein (LDL) receptor. Similarly to apoA-I, apoE4 also has two structural domains (Figure 3c, left), a N-terminal elongated four-helix bundle and a C-terminal, highly α-helical domain of yet unknown structure [40]. Recently, it was reported that apoE4 is not globular but, similar to apoA-I, ellipsoidal, with an axial ratio of ~7:1 [41]. A salt bridge between Arg61 in the N-terminal domain and Glu255 in the C-terminal domain presumably stabilizes an extended helical structure at the C-terminus to support the interaction with large VLDLs [26,42].

Adsorption of apoE4 causes the largest increase in *R*_H_ for the three proteins studied here, by ~6 nm (Figure 2c, Table 1). Unlike the other two proteins, apoE4 has an extended, positively charged surface patch on its N-terminal domain that seems predestined to bind to the negatively charged NPs (Figure 3c). Related to the assumed position of the C-terminal α-helix, the patch is located almost on the opposite side of the four helix bundle, as indicated in Figure 3c (right). An electron paramagnetic resonance study of apoE4 has implied that the C-terminal domain forms a long α-helix that is arranged parallel to the helix bundle at a distance of ~2 nm [43]. If we assume that the four-helix bundle of apoE4 lies flat on the NP surface, binding with its positively charged patch, and if we add the typical diameter of a single α-helix separated by 2 nm, we obtain an overall thickness of 5–6 nm for the protein corona, which closely matches the observed Δ*R*_H_ (Figure 2c, Table 1). About 65 apoE4 molecules will attach to the NP upon complete formation of the protein corona (Table 1).

### Protein binding affinity

The apolipoproteins differ in their binding affinities for the negatively charged FePt NPs by almost four orders of magnitude, with *K’*_D_ (apoE4) = 0.021 ± 0.003 µM and *K’*_D_ (apoA-I) = 140 ± 60 µM (Table 1). HSA has an intermediate *K’*_D_ of 9.9 ± 4.7 µM. The affinities can be correlated with the surface potentials. The high affinity of apoE4 to the negatively charged NPs most likely arises from Coulomb interactions involving the large patch of positive charge of apoE4 (Figure 3c). The positively charged patch on the HSA surface is less pronounced (Figure 3a) and, consequently, the binding affinity is greatly reduced. For apoA-I, there is only a weak area of positive surface potential (Figure 3b), consistent with the low affinity toward the NPs.

For HSA binding to FePt NPs, we have previously reported a Hill coefficient *n* < 1 [11], which is indicative of anti-cooperative binding, meaning that the binding affinity effectively decreases as more HSA molecules adsorb onto the NPs. This finding can be explained by mutual repulsion of the HSA molecules on the NP surface. Note that HSA exists in blood serum in high concentrations and, thus, should not have a tendency to aggregate. For apoA-I, we found *n* = 1, the non-cooperative case, whereas apoE4 was observed to bind to the NPs in a cooperative manner, with *n* = 1.4. This result may be related to the known tendency of apoE4 to form oligomers in solution [41]. Apparently, apoE4 molecules have interfaces by which they can mutually exert attractive interactions. Consequently, a cooperative effect of apoE4 binding to NPs can be explained by the additional stabilization of an apoE4 molecule on the NP in the presence of a neighboring apoE4 molecule.

## Conclusion

By using 2fFCS, we have quantitatively analyzed the adsorption of three blood serum proteins onto FePt NPs. All three proteins gave rise to a well-defined increase in NP size upon binding. The thickness of the protein corona can be related to a particular orientation of the protein, based on the knowledge of its molecular structure. For apolipoproteins, this result is rather intriguing because they are very flexible and are known to undergo large structural changes upon lipid binding [44]. We have shown that the widely different binding affinities of the three proteins can be related to the presence of positively charged surface patches on the proteins. It is unlikely that the surface charge distribution will be similar if the protein structure changes markedly upon binding. Consequently, the observation of positively charged patches on the proteins, which appear to mediate the interaction with our negatively charged NPs, further supports our view that the apolipoproteins do not significantly change their structures upon NP binding. However, the evidence from 2fFCS presented here is rather indirect. In future studies, we shall employ more structure-specific spectroscopic methods such as single-particle FRET, which may yield more detailed insights into the structural properties of the protein corona surrounding NPs.

## Experimental

### Sample preparation

FePt NP cores were synthesized according to published protocols [45] and coated with an amphiphilic polymer synthesized from dodecylamine and poly(isobutylene-*alt*-maleic anhydride). They carry carboxylic acid groups on their surfaces, making them water-soluble. The polymer shell was labeled with the amino-modified fluorescent dye DY-636 (Dyomics, Jena, Germany).

2fFCS measurements were performed in PBS buffer, pH 7.4 (Dulbecco’s PBS without Ca^2+^ and Mg^2+^, PAA Labs, Cölbe, Germany). All proteins were purchased from Sigma (Sigma–Aldrich, St. Louis, MO). NP solutions at (1 ± 0.5) nM were mixed with equal volumes of solutions containing the proteins at varying concentrations. Because of the high affinity of apoE4 to the FePt NPs, the NP concentration was reduced to (0.1 ± 0.05) nM to ensure that only a small fraction of apoE4 proteins was bound to the NPs even at the lowest protein concentrations studied. All protein solutions were prepared by repeated dilution of a single stock solution. The apoE4 dilution series was prepared 2 h before mixing with the NPs to allow the sample to equilibrate between monomers and oligomers [41]. The experiments with apoA-I were limited to below 300 µM because of aggregation problems at higher concentrations. The lack of data in the high-concentration range (Figure 2b) was compensated by enhanced data statistics at the lower concentrations. The NPs were incubated with the proteins for 10 min prior to the measurement.

### 2fFCS setup

**Figure 4:**
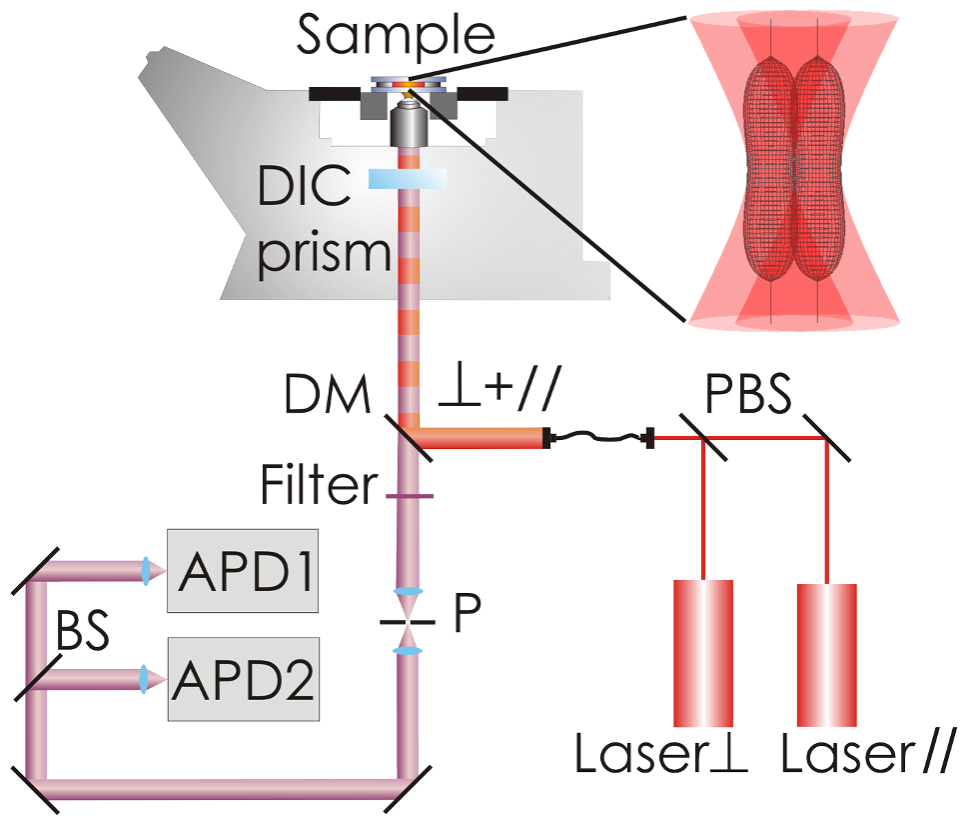
Schematic of the 2fFCS system. DM: dichroic mirror; BS: beam splitter; PBS: polarizing beam splitter; APD: avalanche photo diode; P: pinhole.

The 2fFCS setup is based on a time-resolved confocal microscopy system (Microtime 200, PicoQuant, Berlin, Germany). Instead of using a single excitation laser, the light from two identical, orthogonally polarized pulsed 640 nm diode lasers (LDH-P-C-640B, Picoquant, Berlin, Germany) was combined by a polarizing beam splitter (Figure 4). The lasers were pulsed alternately, each with a repetition rate of 20 MHz, so that the time lag between successive pulses was 25 ns and, thus, much longer than the fluorescence lifetime of the DY-636 dyes (~0.5 ns [46]). Both lasers were coupled into a polarization retaining optical fiber. After exiting the fiber, the light was again collimated into a parallel light beam consisting of a train of laser pulses with alternating orthogonal polarizations. The beam was passed through a dichroic mirror (470/635 nm) and then a Nomarski DIC prism (U-DICTHC, Olympus, Hamburg, Germany), which deflects the laser pulses into two different directions, according to their polarization, into the objective (UPLSAPO 60XW, Olympus) of the inverted microscope (IX71, Olympus). Two overlapping excitation foci (Figure 4) were generated in the sample, with a lateral shift of 404 nm in our setup. The fluorescence light was collected by the same objective and passed through the prism and the dichroic mirror. After the pinhole (150 µm), the light was collimated, split and focused onto two avalanche photodiode (APD) detectors (SPCM-AQR-13, Perkin Elmer, Rodgau, Germany). A single-photon counting card (HydraHarp 400 picosecond event timer und TCSPC module, PicoQuant) recorded the detected photons with picosecond time resolution, so that the photons could be assigned unambiguously to the excitation in one or the other of the two foci. Autocorrelation functions for each detection volume as well as cross-correlation functions between the two detection volumes were calculated from the photon arrival time traces.

### Data collection

For data collection, a few microliters of the sample solution were placed between two standard microscope cover slips separated by a 200 µm thick mylar foil with a 1 mm wide channel for the sample solution in the middle.

Samples were illuminated continuously for 8 min, with the power of each laser adjusted to 3 µW. For NP concentrations of 1 nM (0.1 nM), ~10,000 (1,000) single molecule bursts were analyzed. The temperature was measured during the experiments and accounted for in the determination of the diffusion coefficient, *D*, according to the Stokes–Einstein relation,

[6]
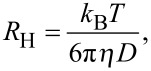


with hydrodynamic radius *R*_H_, Boltzmann constant *k*_B_, temperature *T*, and viscosity *η*. Three independent series of measurements were taken and averaged.

### Data analysis

In conventional FCS, the MDF is typically approximated by a three-dimensional Gaussian profile. However, this assumption is rather crude. In 2fFCS, data fitting is facilitated by a new, semi-empirical two-parameter model describing the MDF [25]. In each lateral (*x,y*-)plane along the optical axis, *z*, the MDF is, for both foci, modeled by a two-dimensional Gaussian function, *U*(*x,y*), of width *w*(*z*) and amplitude *κ*(*z*)/*w*^2^(*z*),

[7]
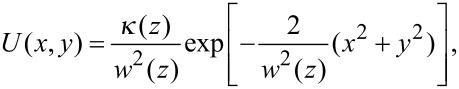


with

[8]
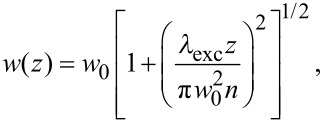


[9]
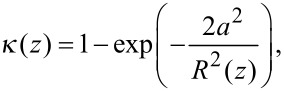


[10]
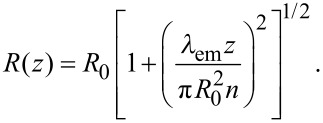


In these equations, *λ*_exc_ and *λ*_em_ are the excitation and the center emission wavelengths, respectively; *n* is the refractive index of the immersion medium and *a* is the radius of the confocal aperture divided by the magnification. *R*_0_ and *w*_0_ are a priori unknown model parameters that are determined by the fit.

As the emitted photons are registered as a function of time, they can be assigned to one of the two foci. Therefore, three correlation functions can be calculated from the data from each of the two foci, that is, the two auto-correlation functions and the cross-correlation function. Actually, a cross-correlation between two detectors is also performed to calculate the autocorrelation functions, so to avoid afterpulsing artifacts of the APDs. All three correlation functions are fitted globally, according to

[11]
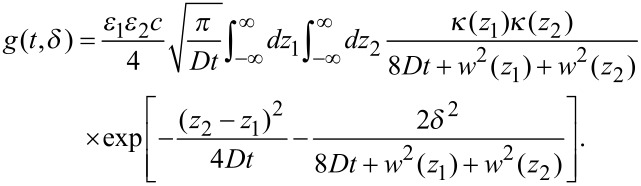


The coefficients *ε*_1_ and *ε*_2_ take the proper weighting of the two polarization channels, due to the different excitation powers and detection efficiencies, into account. For the auto-correlation curves, the spatial separation of the two foci, *δ*, is set to zero, and *ε*_1_*ε*_2_ is replaced by *ε*_1_^2^ or *ε*_2_^2^.

The correlation analysis was performed with the SymphoTime software (PicoQuant). FCS experiments are notoriously sensitive to the presence of large aggregates, therefore, those parts of the time traces that showed excessively high intensities were excluded from the correlation analysis. Changes in viscosity due to the increasing protein concentration were taken into account by using a linear approximation for the contribution of the solute to the solution viscosity, based on the intrinsic viscosity of HSA of 4.2 cm^3^/g, as specified by the supplier (Sigma–Aldrich, St. Louis, MO), and of apoA-I (9.2 cm^3^/g) [47]. The viscosity change due to apoE4 has, to the best of our knowledge, not yet been determined. However, the viscosity effect of apoE4 is minimal in our experiments because its high affinity to the NPs required the use of lower concentrations.
